# Improved Self-Organizing Map-Based Unsupervised Learning Algorithm for Sitting Posture Recognition System

**DOI:** 10.3390/s21186246

**Published:** 2021-09-17

**Authors:** Wenyu Cai, Dongyang Zhao, Meiyan Zhang, Yinan Xu, Zhu Li

**Affiliations:** 1 College of Electronics and Information, Hangzhou Dianzi University, Hangzhou 310018, China; zhaody@hdu.edu.cn (D.Z.); lz1126@hdu.edu.cn (Z.L.); 2 College of Electrical Engineering, Zhejiang University of Water Resources and Electric Power, Hangzhou 310018, China; xunancyg@163.com

**Keywords:** sitting posture recognition, flexible pressure array, self-organizing map, unsupervised self-learning algorithm

## Abstract

As the intensity of work increases, many of us sit for long hours while working in the office. It is not easy to sit properly at work all the time and sitting for a long time with wrong postures may cause a series of health problems as time goes by. In addition, monitoring the sitting posture of patients with spinal disease would be beneficial for their recovery. Accordingly, this paper designs and implements a sitting posture recognition system from a flexible array pressure sensor, which is used to acquire pressure distribution map of sitting hips in a real-time manner. Moreover, an improved self-organizing map-based classification algorithm for six kinds of sitting posture recognition is proposed to identify whether the current sitting posture is appropriate. The extensive experimental results verify that the performance of ISOM-based sitting posture recognition algorithm (ISOM-SPR) in short outperforms that of four kinds of traditional algorithms including decision tree-based (DT), K-means-based (KM), back propagation neural network-based (BP), self-organizing map-based (SOM) sitting posture recognition algorithms. Finally, it is proven that the proposed system based on ISOM-SPR algorithm has good robustness and high accuracy.

## 1. Introduction

As everyone knows, sedentary behavior is becoming more common, while most white-collar workers spend a lot of time sitting down in front of computer and not moving. The ensuing problems are inevitable with the widespread existence of sedentary lifestyles, and the harm to human health is gradually increasing [1]. Modern medical studies have shown that long-term slouching has a direct influence on the cervical spine unevenness, oppressing the stretching of the neck muscle, etc. [2]. The link between a poor sitting posture and bad health consequences is growing with clear evidence, including obesity, cardiovascular and metabolic diseases, and even cancer. In addition, sedentary behavior with abnormal posture has an impact not only on physical health but also on psychosocial health. Sanchez-Villegas et al. [3] provide a positive correlation between sedentary behavior and the risk of developing a mental disorder, with those at the highest level of the sedentary index having a 31% higher risk of mental disorder when compared with less sedentary individuals. Among them, inappropriate sitting postures are the main causes for the above health problems [4,5]. As a result, the innovative research on sitting posture recognition system is of great significance to the sedentary crowd’s health.

According to the existing literature, two types of methods are often used to distinguish sitting posture: wearable [6,7,8,9,10] and non-wearable [11,12,13,14]. The detection sensors of the former are mainly relying on an accelerometer, inertial sensor, and gyroscope [6]. In this approach, wearable sensors are attached either to the user’s back [7], shoulders [8], or legs [9] to collect human motion state. Hence it may be inconvenient and uncomfortable to wear such electronic devices for a normal person [10], leading to narrow applicability and less maneuverability. The latter can be further divided into two categories, the first method is based on machine vision, and the other is based on simple pressure distribution [11]. The machine vision-based posture recognition approach gets information from cameras covering sitting people, and then identifies the sitting posture by image processing [12] or machine learning [13]. However, it is difficult to ensure robustness depending on the light conditions and shooting angles [14], and it also involves some privacy issues. The simple pressure distribution-based approach is to place sensors at some specific locations of a chair or backrest and use feature information collected by these sensors to recognize sitting postures [15]. This method may be uncomfortable and low accurate. Experimental results of [16] shows that the overall accuracy of this method is 85.9%, and the accuracy rate reaches 83.33% in [17]. As an exploration and improvement, we study the method of sitting posture recognition based on the flexible array pressure sensor for real-time and accurate sitting posture recognition. Many algorithms have been applied to sitting posture recognition in existing papers, Benocci et al. [18] use the *k*-nearest neighbor (*k*NN) for sitting postures recognition, the derived accuracy degree is 92.7%. Liu et al. [19] apply the convolutional neural network (CNN) to the sitting posture recognition and can achieve 95.5% accuracy. Kim et al. [15] also apply CNN to the sitting posture recognition and compare it with decision tree (DT), multinomial logistic regression (MLR), etc. The average accuracy is 95.3%. Unfortunately, the complexity of the CNN algorithm is relatively high, so it is difficult to apply it in real-time recognition. We use SOM algorithm as the basic algorithm for the recognition of sitting postures. The simple network structure of SOM ensures the real-time recognition of the system for sitting postures. The data collected by our self-made data acquisition system is similar for the same sitting posture. Considering the characteristics of sitting posture data, the output speed of the proposed system using SOM algorithm will be fast and accurate.

The main contributions of this paper are:

1. This paper proposes one novel sitting posture recognition system based on a flexible array pressure sensor and overcomes the dependence on the environment of the sitting posture recognition method based on machine vision.
2. This paper introduces the SOM algorithm for sitting posture recognition for the first time, and an improvement of SOM issued to optimize connected weights, therefore, it solves the problem of the low recognition rate of the traditional method based on simple pressure distribution.

The rest of this document is organized as follows. Section 2 proposes detailed system framework of sitting posture recognition. In Section 3, SOM based sitting posture pressure recognition algorithm is described in detail. Moreover, an improved SOM considering the optimization of weights is proposed to overcome the limitations of SOM network structure and to achieve better results. Experimental results are presented in Section 4, and a summary of the whole paper is made in Section 5.

## 2. System Model

One novel sitting posture recognition system composed of flexible array pressure sensor and cloud platform is designed in this paper. The whole system consists of three main components: pressure acquisition circuit, cloud platform service and analysis and monitoring software. The pressure acquisition circuit is responsible for pressure data collection, and then transmits the pressure data to the cloud platform according to the user’s wishes, while the cloud platform saves the data and transfers real-time data to the analysis and monitoring software, and finally receives the recognition results. The analysis and monitoring software is responsible for the visualization of pressure distribution of sitting postures, and the implementation of some sitting postures recognition algorithms. During the process of data analysis, we constructed a variety of sitting posture recognition algorithms, and an improved SOM algorithm for postures classification is discussed. After testing and analyzing, the sitting posture recognition algorithm based on improved SOM (ISOM-SPR) has strong robustness and high accuracy compared with other existing algorithms.

**Figure 1:**
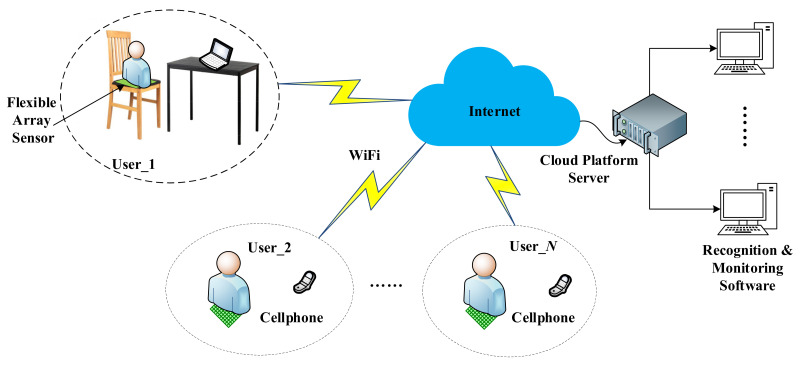
System framework.

The framework of the proposed sitting posture recognition system is shown in Figure 1. Each user is sitting on a special chair equipped with a flexible array pressure sensor, and we self-made data acquisition system to collect real-time pressure values and users have the power to choose whether to transmit it to the Internet. If certain user agrees, his sitting posture information would be uploaded to the cloud platform for better management. Users can check information about their sitting postures via their cell phones or computers, the recognition and monitoring software provides a visual display of real-time data.

**Figure 2:**
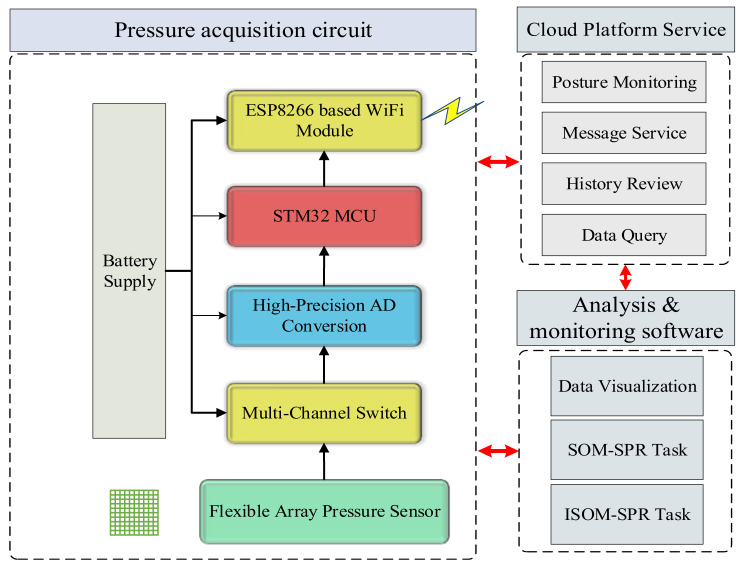
The block diagram of the sitting posture recognition system.

The block diagram of the sitting posture recognition system is described in Figure 2, which is composed of three main parts: pressure acquisition circuit, cloud platform and analysis and monitoring software.

**Figure 3:**
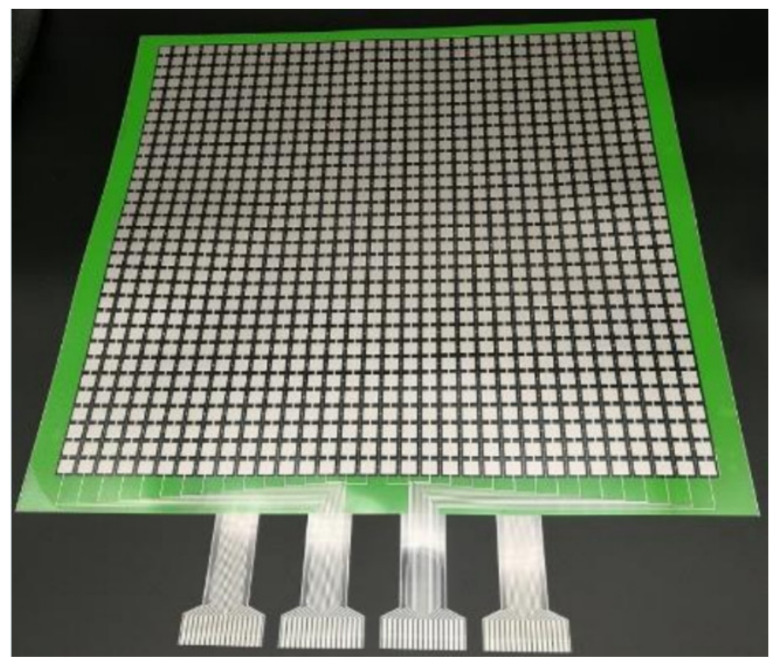
Flexible array pressure sensor.

The pressure acquisition circuit mainly includes a STM32 MCU based data acquisition board and a flexible array pressure sensor in Figure 3. The array pressure sensor is a large-area flexible pressure sensor. The sensor has a total of four sets of interfaces, each interface has 16 pins, so there are a total of 32 row pins and 32 column pins. The pins are designed to achieve efficient data acquisition with the minimum number of pins. There are 1024 independent induction units distributed within a 400 mm × 400 mm square, and each induction point is 8.5 mm × 8.5 mm in size. The size of sensor array is designed by combining the size of actual seat and area of contact between buttocks and seat when users sit down. One point’s resistance of this strain gauge-designed sensor will change when the pressure it receives changes.

**Figure 4:**
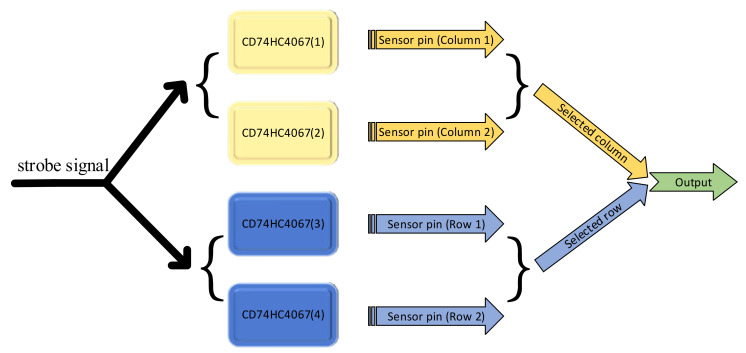
Schematic diagram of analog switch connection.

Since the applied flexible array pressure sensor has 1024 independent sensing point, we could not have access to all the points at one time. We acquire the data of one sensing point with multi-channel switch CD74HC4076. We provide a strobe signal for multi-channel, only one row pin and one column pin are strobed at the same time as shown in Figure 4. The output signal at this moment is the pressure information of the sensor node corresponding to the row and column.

**Figure 5:**
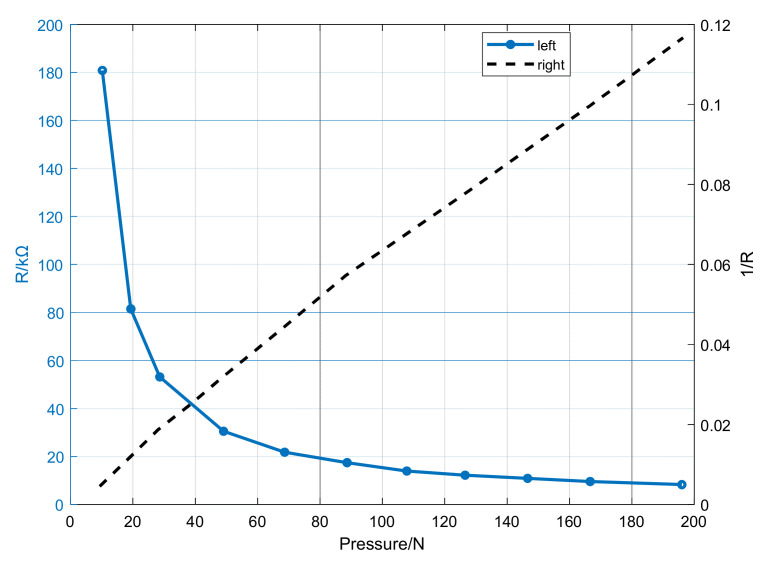
The characteristic curve of array pressure sensor. The left shows the pressure is directly proportional to the resistance 1/*R*; The right shows the pressure is inversely proportional to *R*.

The pressure is then obtained by high-precision A/D convertor [20]. The resistance of each point is *R_ij_*. Considering the different softness of different seats, we first filtered the collected data in the process of data collection. Hence, the resistance change caused by the sensor deformation is much less than that caused by human body pressure. We set a threshold, and only data above that threshold will be collected. The relational expression between output voltage *u_o_* and resistance *R_ij_* of each induction point is illustrated in Equation (1).

(1) $$u_{o}=V_{ref}\times \frac{R_{ij}}{R_{ij}+R_{IM}},(i,j=1,2,\ldots ,32),\;$$

where *V_ref_* is 5 V, the impedance matching resistance *R_IM_* is 200 kΩ, so it has both good linearity and the relatively wide voltage range. The change of resistance *R_ij_* is determined by the pressure at the corresponding induction point, the relationship between pressure *p_ij_* and resistance *R_ij_* is shown in Figure 5, which could be expressed as $p_{ij}=1690/R_{ij}-3.379$ with actual measurement.

**Figure 6:**
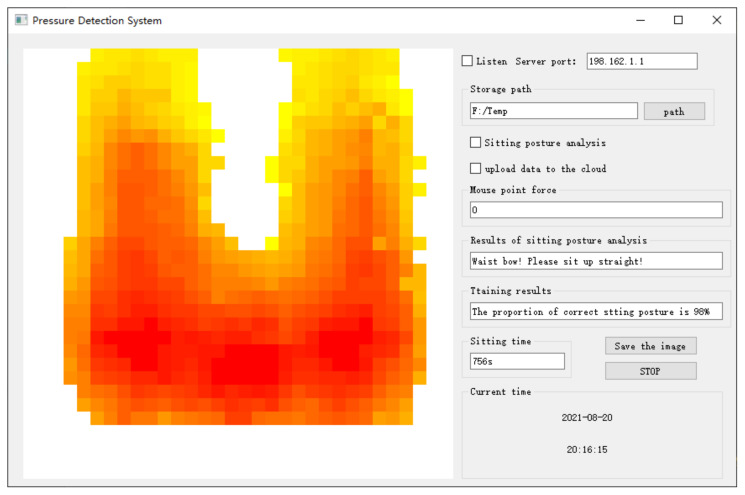
The software interface of analysis and monitoring application.

**Figure 7:**
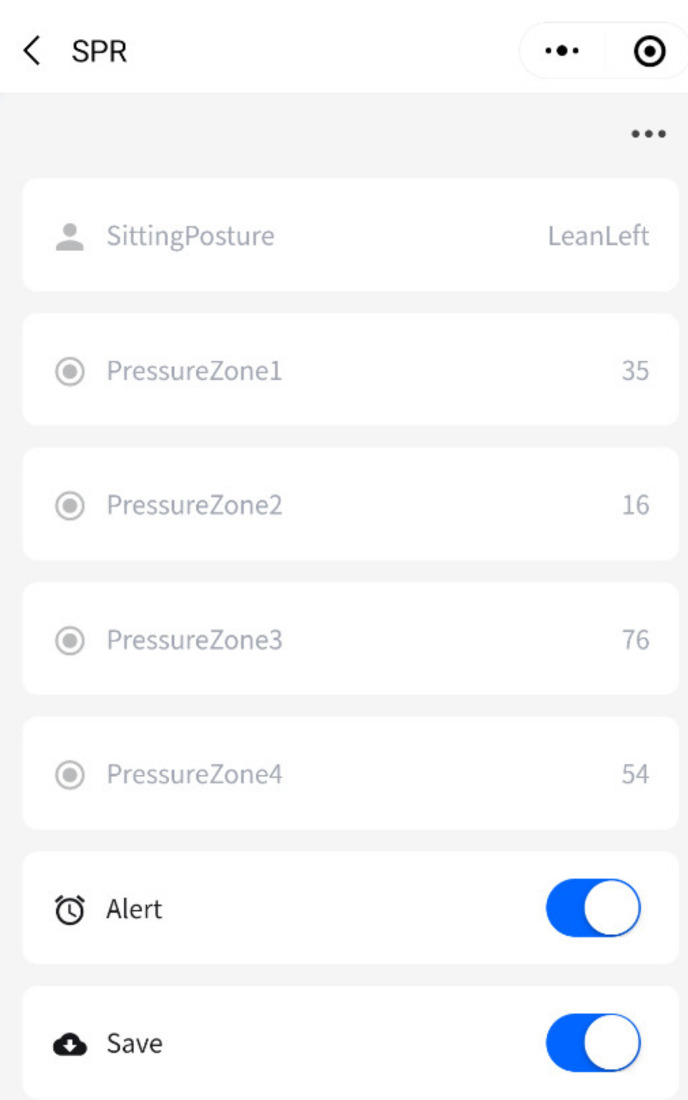
The interface on the phone.

The analysis and monitoring software is mainly responsible for the implementation of some sitting postures recognition algorithms, real-time display of pressure distribution. Users will be alerted if they are sitting with inappropriate postures, it also alerts users if they are sitting with some posture for too long time. The pressure detection system interface is shown in Figure 6. Users can also save the current sitting posture pressure map. The entire system will still work without an Internet connection and the data will only be stored on the user’s PC, not available from the phone. Each pressure acquisition circuit has the ability of transmitting sensory data to the cloud platform service with Wi-Fi. The cloud platform service will receive and save these sensory data for each user in a confidential manner. Users can also check the information through their cell phones if the users agree to upload the data to the cloud platform. It is convenience for users to view records anytime and anywhere. In this case, users such as the elderly or patients with cervical Spondylosis can get better help and comprehensive supervision. The interface on the phone is shown in Figure 7. We divided the whole sensor array into four regions, namely, upper right, upper left, lower right and lower left, corresponding to pressure zone 1–4, respectively. It also helps the user numerically check how he or she is sitting.

**Figure 8:**
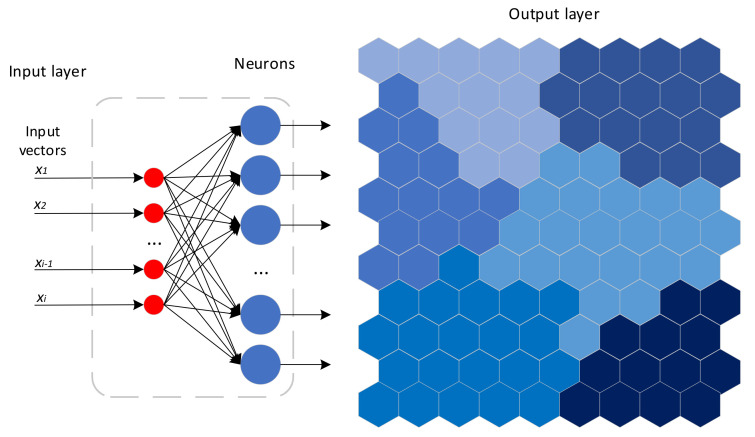
The SOM network structure diagram.

To improve classification accuracy, the self-organizing map algorithm [21], one unsupervised artificial neural network method, is introduced into the designed recognition algorithm of sitting postures. The self-organizing map (SOM) network structure has two layers: the input layer and the output layer. The SOM generates a low-dimensional, discrete map by learning data in the input space. The number of neurons in the input layer is determined by the dimension of the input vector $\vec{x}=\left(x_{1},x_{2},\ldots ,x_{i}\right)$. The structure diagram of SOM and classification results are described in Figure 8. With self-learning by SOM, the more similar the data, the more likely it is fall into the same category.

## 3. SOM Based Sitting Posture Recognition Algorithm

**Figure 9:**
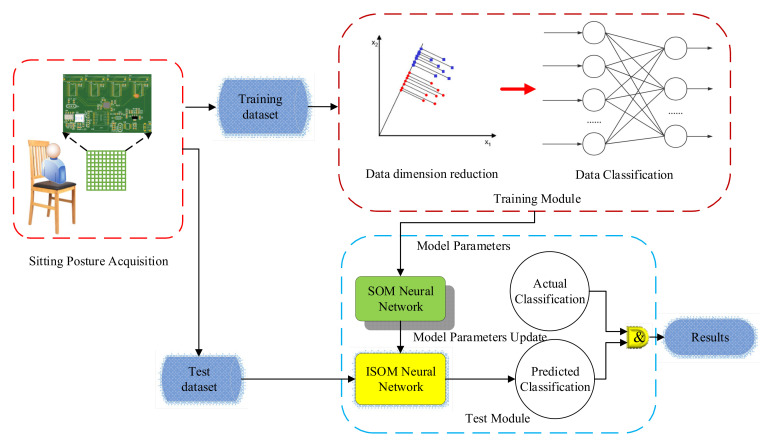
The principle of proposed algorithm.

**Figure 10:**
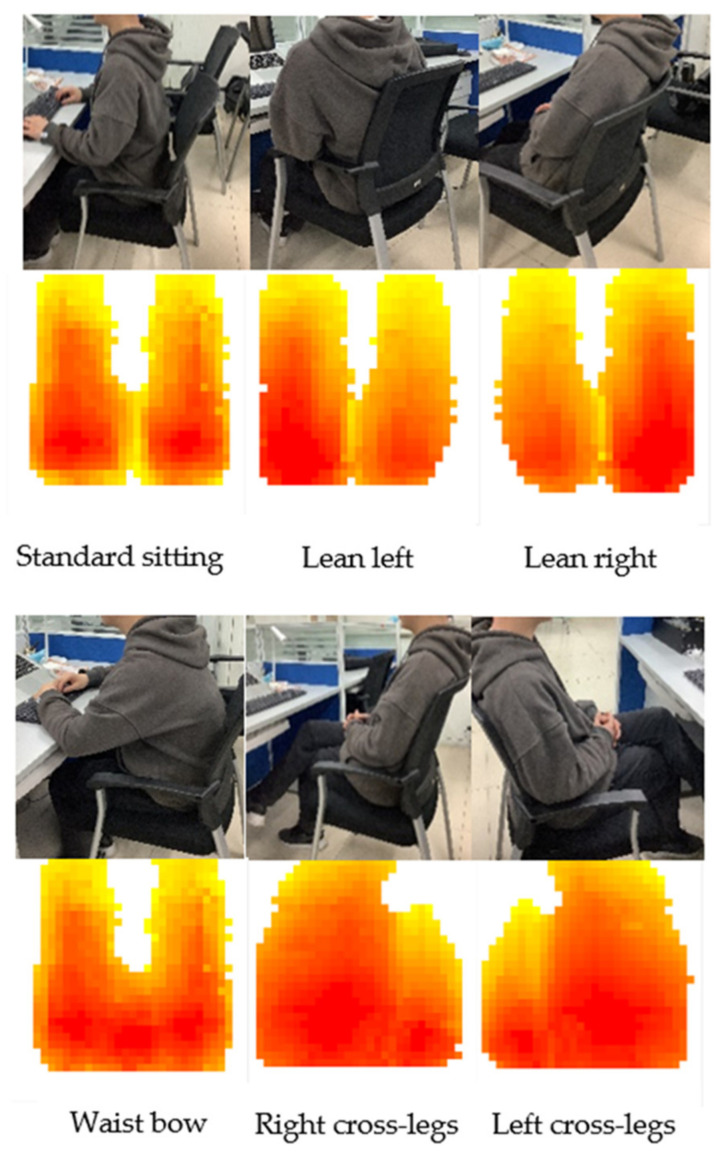
Sitting posture and pressure distribution.

The principle of the sitting posture recognition process is demonstrated in Figure 9. The original data collected by the pressure acquisition circuit are taken as the original dataset. Each set of data is a matrix with rows and columns of 32. Due to a large amount of data, the sensible first step is to reduce the dimension of data. However, the characteristics of the data should be preserved as much as possible while the data volume is reduced. After the data are reduced in dimensionality, it is classified using the SOM algorithm. SOM algorithm, on the one hand, can put similar data in the same category, on the other hand, maps the input data into two-dimensional discrete graphics in the output layer. These characteristics apply well to the data processing of sitting posture. The collected data are divided into training part and testing part. The actual sitting posture of the human body corresponding to the pressure distribution received by the pressure sensor array is described in Figure 10. Six sitting postures, namely standard sitting, lean left, lean right, waist bow, right cross-legs, and left cross-legs [22] are common sitting postures, so classification and detailed data analysis are carried out for these six sitting postures.

### 3.1. Dimensionality Reduction for Original Data

The number of neurons in the output layer is positively correlated with the number of categories of training samples. If the number of neurons is less than that of categories, it is not enough to distinguish all the categories, hence the results of training are bounded to combine the similar classes into one. On the contrary, if there are more neurons than categories, it is possible to divide them excessive or to have “dead nodes”, i.e., one node never wins and is far away from other winning nodes during the training process, so their weights will never update.

The original pressure distribution data area matrix with 32 rows and 32 columns as following, so the number of input nodes in this network is 1024 theoretically. As a time-varying matrix, *P* is changing over time. The following descriptions are all in the state of time t. For the sake of clarity, the variable t is omitted.

(2) $$P=\left(\begin{array}{cccccc} p_{1,1} & p_{1,2} & . & . & . & p_{1,32}\\ p_{2,1} & p_{2,2} & . & . & . & p_{2,32}\\ . & . & . & & & .\\ . & . & & . & & .\\ . & . & & & . & .\\ p_{32,1} & p_{32,2} & . & . & . & p_{32,32} \end{array}\right).$$

Excessive data input may introduce unnecessary noises, increase computation complexity, thus slow down the speed of data processing. Therefore, we pre-process the input data and compress the data by principal component analysis (PCA) [23]. $P=\left(P_{1},P_{2},\ldots ,P_{32}\right)$ can also be thought of as 32-dimensional vectors, each of which has 32 elements, $P_{k}={\left(p_{1,k},p_{2,k},\ldots ,p_{32,k}\right)}^{T}.$ The covariance matrix is defined as follows:

(3) $$R=\left(\begin{array}{cccccc} r_{1,1} & r_{1,2} & . & . & . & r_{1,32}\\ r_{2,1} & r_{2,2} & . & . & . & r_{2,32}\\ . & . & . & & & .\\ . & . & & . & & .\\ . & . & & & . & .\\ r_{32,1} & r_{32,2} & . & . & . & r_{32,32} \end{array}\right).$$

Every member $r_{mn}$ in the covariance matrix is a covariance between $p_{m}$ and $p_{n}$, the calculation method [24,25] is as follows:

(4) $$r_{mn}=cov(p_{m},p_{n})=\frac{{\sum_{k=1}^{m}(p_{k}-\bar{p}}_{m})(p_{k}-{\bar{p}}_{n})}{m-1},(m,n=1,2,\ldots ,32).$$

The eigenvectors $\varphi_{1},\varphi_{2},\varphi_{3},\ldots ,\varphi_{32}$ and their corresponding eigenvalues of the matrix *R* are calculated. If the eigenvectors are over-reserved, the noise will also be retained, and the amount of data will not be reduced significantly, thus the calculation speed will be negatively affected. However, the difference between the samples will not be observed if too few eigenvectors are selected. After calculation and analysis, the top 8 eigenvectors were selected without affecting the characteristics of the data, and the calculation speed of classification is improved at the same time. The 8 eigenvectors corresponding to the 8 largest eigenvalues are constructed into a matrix $A=\left(\varphi_{k}|\varphi_{k}\in Top_{8}\left(\varphi_{i}\right),i=1,2,\ldots ,32\right)$. Finally, the data matrix *P_r_* after dimensionality reduction is calculated as follows:

(5) $$P_{r}=PA.$$

*P_r_* is a 32 by 8 matrix, the amount of data has been reduced from 1024 to 256.

(6) $$P_{r}=\left(\begin{array}{cccccc} p_{r}{}_{1,1} & p_{r}{}_{1,2} & . & . & . & p_{r}{}_{1,8}\\ p_{r}{}_{2,1} & p_{r}{}_{2,2} & . & . & . & p_{r}{}_{2,8}\\ . & . & . & & & .\\ . & . & & . & & .\\ . & . & & & . & .\\ p_{r}{}_{32,1} & p_{r}{}_{32,2} & . & . & . & p_{r}{}_{32,8} \end{array}\right).$$

### 3.2. Data Classification

PCA is a traditional data compression method under the condition of retaining data characteristics to the maximum extent. After the data arecompressed, the noise will be reduced, and the calculation speed of the SOM network will be improved.

The elements of the matrix *P_r_* are connected from head to tail to construct the input vector of the SOM network. Each vector in the input layer has 256 elements. The input vector can be written as ${\vec{p}}_{ri}=\left\{{\vec{p}}_{ri}:{\vec{p}}_{ri}=\left(p_{ri\;1,1},\;p_{ri\;1,2},\ldots ,p_{ri\;1,8},p_{ri\;2,1},\;p_{ri\;2,2},\ldots ,p_{ri\;2,8},\ldots ,p_{ri\;32,1},\;p_{ri\;32,2},\ldots ,p_{ri\;32,8}\right),m=1,2,\ldots ,32,\;n=1,\;2,\;\ldots ,8,\;i=1,2,\ldots ,M\right\}$, Mdenotes the number of input neurons, which is the number of training samples, the connected weights between input neuron *i* and *j* in the competitive layer can be written as $\;{\vec{w}}_{j}=\left\{{\vec{w}}_{j}:{\vec{w}}_{j}=\left(w_{j1},w_{j2},\ldots ,w_{j256}\right),j=1,\ldots ,N\right\}$, where *N* is the number of output neurons, since the output layer is designed to have 6 nodes, so *N* is set to 6.

The first step is to normalize the selected samples and weights as Equation (7) and Equation (8) [26]. Normalization eliminates the adverse effects caused by singular samples and different data sizes, helps to improve the solution convergence speed, and improves the efficiency of model training.

(7) $${\vec{\overset{-}{p}}}_{ri}=\frac{{\vec{p}}_{ri}}{\left\|{\vec{p}}_{ri}\right\|},\left(i=1,2,\ldots ,M\right).$$

(8) $${\vec{\overset{-}{w}}}_{j}=\frac{{\vec{w}}_{j}}{\left\|{\vec{w}}_{j}\right\|},\left(j=1,2,\ldots ,N\right).$$

The Euclidean distance $d_{ij}$ between the input ${\vec{\bar{p}}}_{ri}$ and the weight ${\vec{\bar{w}}}_{j}$ [26] is calculated as following:

(9) $$d_{ij}=\left\|{\vec{\bar{p}}}_{ri}-{\vec{\bar{w}}}_{j}\right\|,(i=1,2,\ldots ,M,j=1,2,\ldots ,N),$$

(10) $$d_{c}=min\left(d_{ij}\right),(i=1,2,\ldots ,M,j=1,2,\ldots ,N),$$

where $\;d_{c}$ is the smallest distance among $d_{ij}$, and the neuron with the minimal distance will be the winner c.

The neighborhood function is used to determine the influence of the winning node on its near neighbor node, hence update range of each node in the winning neighborhood. The most common choice is the Gaussian function [26].

(11) $$h_{ij}=exp\left(-D_{ij}^{2}-2s^{2}\right).$$

(12) $$D_{cj}=\left\|r_{c}-r_{j}\right\|.$$

The Gaussian based winning neighborhood function is shown in Equation (11), where $D_{cj}$ is the distance between neuron *c* and neuron *j*, $\;r_{c}$ denotes the location of winning neuron c, and $r_{j}$ denotes the location of the near neighbor neuron j. $N_{c}\left(t\right)$ refers to the number of neurons centered on the winning neuron, which is determined by the neighborhood function. $N_{c}\left(0\right)$ is the initial neighborhood value.

We use the exponential function to define the wining neighborhood function’s decay rate,

(13) $$\sigma \left(t\right)=\sigma_{0}exp\left(-t/t_{s}\right).$$

The learning rate is $\eta \left(t\right)\in \left(0,1\right)$, the initial value is $\eta_{0}$. The updates are applied for all the training process. Finally, we will iterate the process until the learning rate converges to 0.

(14) $$\eta (t)=\eta_{0}\exp(-t/\tau_{\eta }).$$

The weight is adjusted to all neurons in the winning neighborhood. If one neuron is in the winner’s neighborhood, the weight will change as the first equation of Equation (15); otherwise, the weight will change as the second equation of Equation (15).

(15) $$\left\{\begin{array}{l} {\vec{\bar{w}}}_{ij}\left(t+1\right)={\vec{\bar{w}}}_{ij}\left(t\right)+\eta \left(t\right)h_{ij}\left(t\right)\left({\vec{\bar{p}}}_{ri}-{\vec{\bar{w}}}_{j}\right),j\in N_{c}\left(t\right)\\ {\vec{\bar{w}}}_{ij}\left(t+1\right)={\vec{\bar{w}}}_{ij}\left(t\right),j\notin N_{c}\left(t\right) \end{array}\right..$$

### 3.3. SOM Based Sitting Posture Recognition Algorithm (SOM-SPR)

The arrangement form of neuron nodes in the output layer depends on the needs of practical applications, and the arrangement form should reflect the physical meaning of practical problems as intuitively as possible.

**Figure 11:**
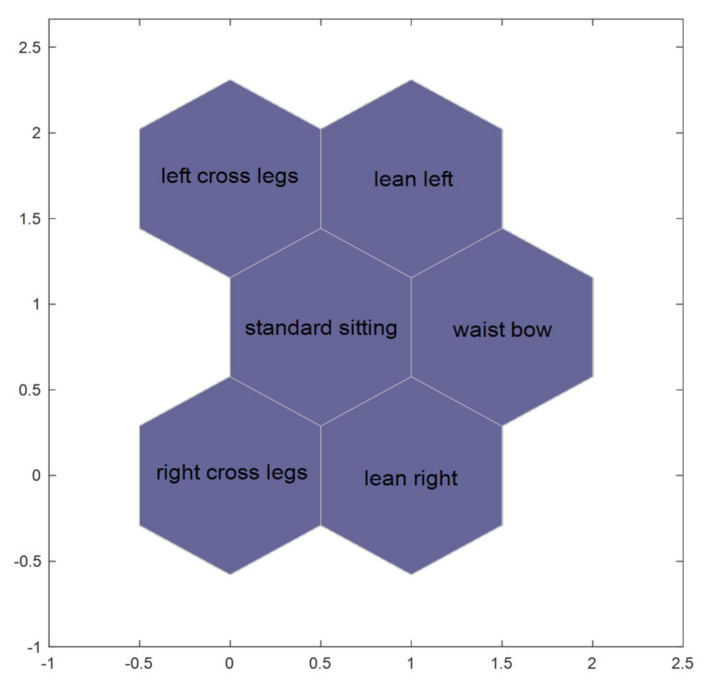
The SOM-SPR topology of the output layer.

Six sitting postures, including left cross legs, right cross legs, lean left, lean right, waist bow, and standard sitting, are arranged to identify, so the number of output nodes is 6. Moreover, the neurons are connected via hexagonal topology [27], as Figure 11. All the data are distributed on a two-dimensional plane. If the posture is similar, the distance will be closer in topology. For instance, the center is “standard sitting”, “left cross legs” and “lean left” are above “standard sitting”. This structure is also in line with human experience and cognition.

**Figure 12:**
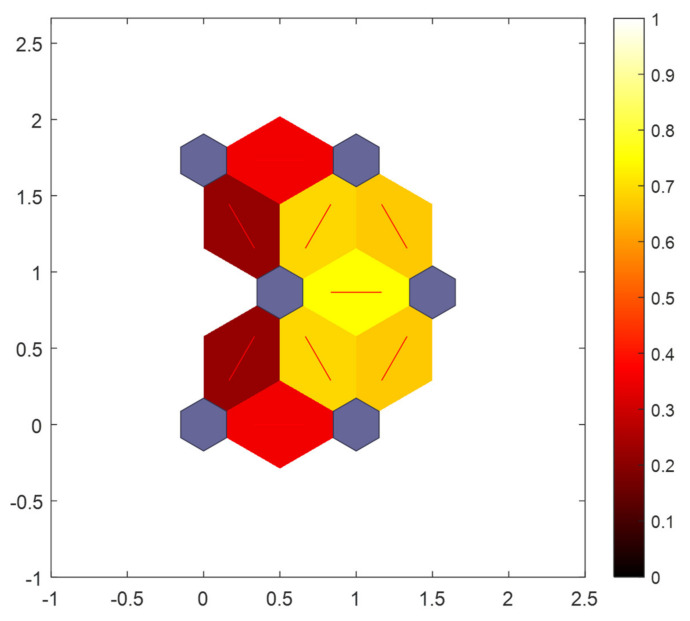
The SOM-SPR neighbor nodes distance.

The results of Figure 12 show that the sitting posture clustering results make two classes with high similarity closer to each other, the more similar the two categories are, the closer the distance is. As shown in Figure 12, the color ranges from red to orange and then to yellow. The lighter the color, the higher the similarity of their features. As a result, the distance between “standard sitting” and “waist bow” is very small, while the distance between “standard sitting” and “waist bow” is very big. In terms of results, the accuracy of SOM-SPR is excellent, however, we have made improvements to the SOM network in pursuit of better results, details in Section 3.4.

### 3.4. Improved SOM Based Sitting Posture Recognition Algorithm (ISOM-SPR)

As we know, SOM algorithm is an unsupervised learning method, which has good characteristics of self-organizing and visualization. However, there are still some apparent limitations, such as the network structure is fixed and cannot change dynamically. Therefore, an improved SOM approach to solve this problem is studied to achieve better recognition results of sitting posture.

Once the SOM-SPR algorithm finished once clustering, the weights will not change. Therefore, we recalculate the Euclidean distances between the input ${\vec{\bar{p}}}_{ri}$ and the fixed weight ${\vec{\bar{w}}}_{j}$.

(16) $$d_{newij}=\left\|{\vec{\bar{p}}}_{ri}-{\vec{\bar{w}}}_{j}\right\|.$$

Hence, the difference $e_{jk}$ between adjacent nodes of the same data is calculated. j and k are the labels of two adjacent nodes, respectively.

(17) $$e_{jk}=\left|d_{newij}-d_{new\mathrm{ik}}\right|,(i=1,2,\ldots ,M,j,k=1,2,\ldots ,N,k\neq j).$$

(18) $$r_{jk}=\frac{e_{jk}}{d_{newij}+d_{newik}}.$$

**Figure 13:**
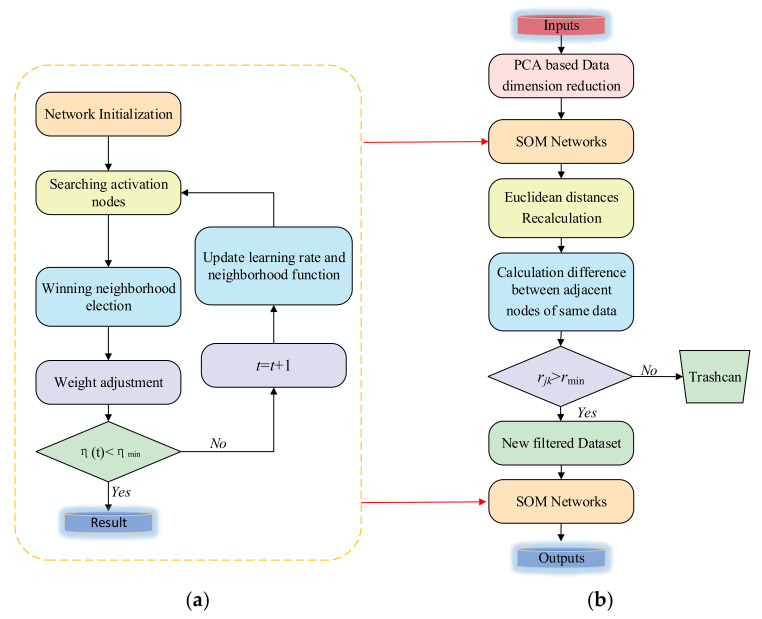
Flow charts of two algorithms. (**a**) Flow chart of SOM-SPR. (**b**) Flow chart of ISOM-SPR.

The degree of differentiation of the same data relative to two nodes is defined as $r_{jk}$. As the number $r_{jk}$ gets bigger, the difference gets bigger and bigger. Onthecontrary, the smaller the $r_{jk}$, the less significant the difference. The corresponding set of data will be removedwhen $r_{jk\;}$ is less than certain threshold value $r_{min}$. The flow charts of SOM-SPR algorithm and ISOM-SPR algorithm are shown in Figure 13.

**Figure 14:**
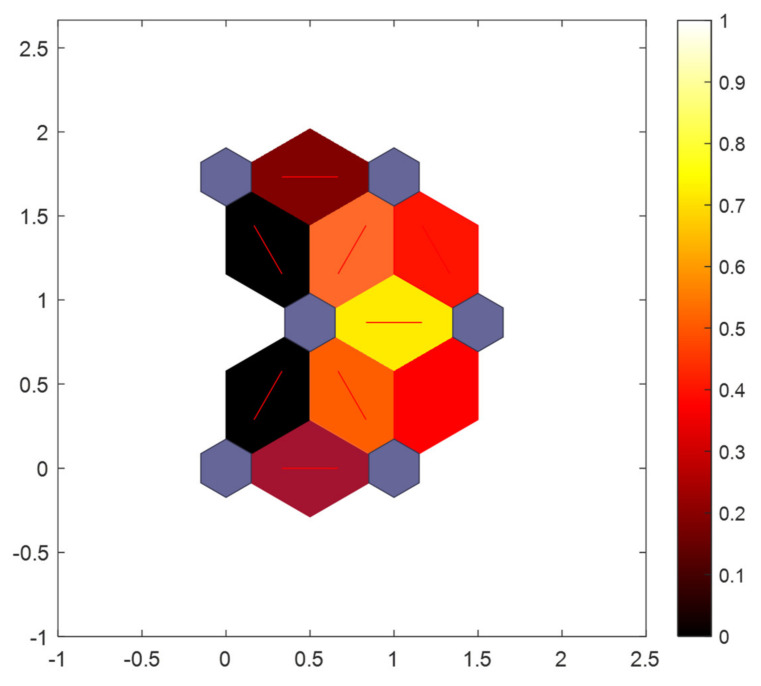
The ISOM-SPR neighbor nodes distance.

After that, we use the retained data to complete the learning process again. In this way, the result characteristics of final clustering will be more obvious. In other words, the distance between nodes in the output layer will be larger, as Figure 14.

The parameters in the training process for SOM-SPR and ISOM-SPR algorithms are listed in Table 1.

## 4. Experiment Results

### 4.1. Data Sets

**Figure 15:**
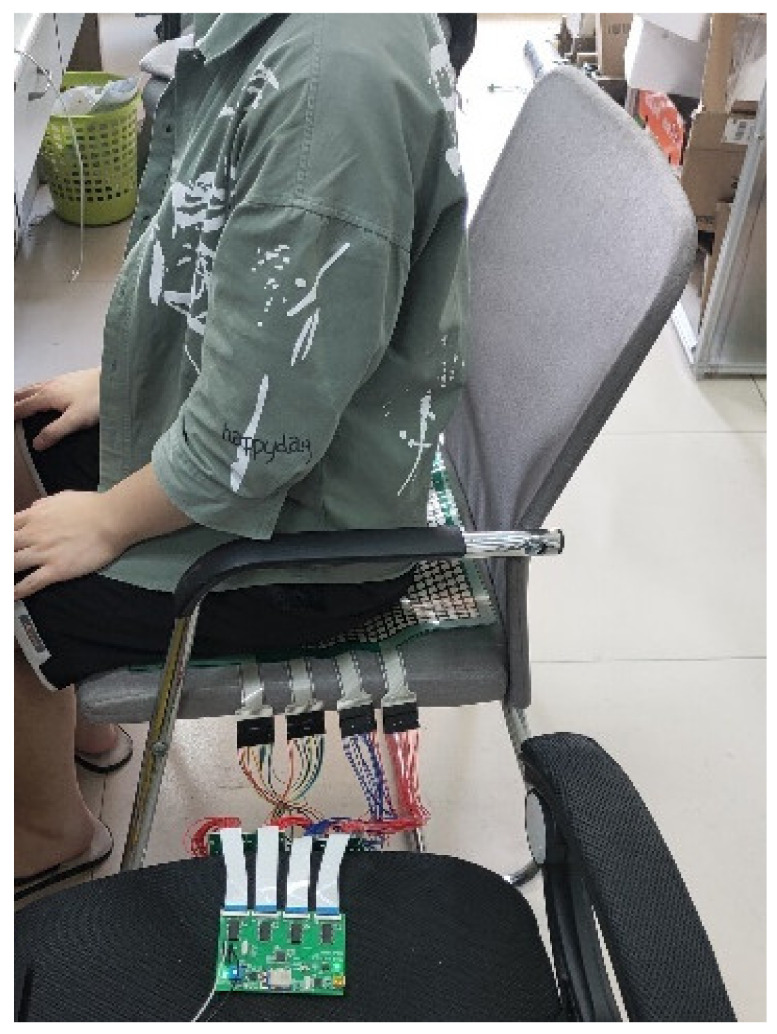
Experimental scene.

**Figure 16:**
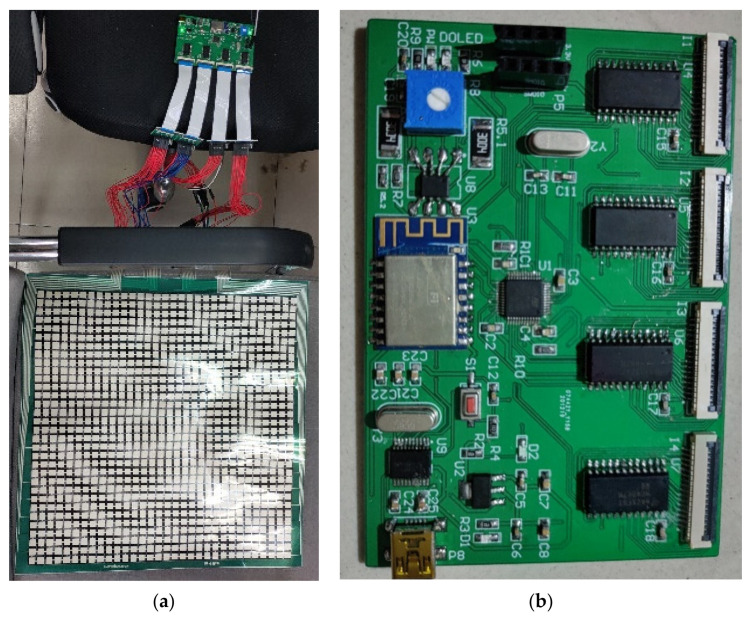
The data acquisition system and circuit. (**a**) The data acquisition system. (**b**) Pressure acquisition circuit.

In our experiments, 40 volunteers participated, including 10 females and 30 males. The heights of the volunteers ranged from 1.58 m to 1.81 m, and the weights ranged from 45 kg to 80 kg. Participants were asked to sit with six different positions, and we recorded data on each participant’s six sitting postures. During the test, the volunteers sat on the sensor as shown in Figure 15. The whole system is shown in Figure 16a, and the self-made data acquisition circuit is exhibited in Figure 16b.

For the sake of clarity, different sitting postures are labeled respectively, left cross-legs as LC, right cross-legs as RC, lean left as LL, lean right as LR, waist bow as WB, and standard sitting as SS. We saved around 30 frames for each pose and had a total of 8000 sets of data to form Dataset 1.

The general idea of training is as follows: firstly, all data samples are divided into 55% as training samples and 45% as test samples. In each sample, the data from the used flexible pressure array sensor are taken as input vectors, and the corresponding sitting posture is taken as the output.

### 4.2. Confusion Metrics

The confusion matrix is a measure used while solving classification problems [28]. It can be applied to binary classification as well as for multi-class problems. A multi-class confusion matrix for “left cross-legs” is shown in Table 2.

(19) $$Accuracy=\frac{TP+TN}{TP+TN+FP+FN}.$$

(20) $$Recall=\frac{TP}{TP+FN}.$$

(21) $$Precision=\frac{TP}{TP+FP}.$$

### 4.3. Experimental Result

In the test phase, we compare five traditional methods for sitting posture recognition, namely Decision tree-based SPR(DT-SPR) [15], *K*-means based SPR (KM-SPR) [29], BP neural network based SPR(BPNN-SPR) [30], SOM network based SPR(SOM-SPR), and the improved SOM network based SPR(ISOM-SPR). Table 3 shows the precision comparable results of different SPR algorithms. It is obvious that the performance of DT-SPR is the worst, ISOM-SPR has the highest average precision.

Table 4 compares the recall rate of different SPR algorithms. For postures LC and RC, three algorithms have a 100% recall rate. The performance of ISOM-SPR is better than all the other algorithms in terms of average recall rate.

Since training data and test data come from the same group of volunteers, there is a certain degree of similarity in the dataset, which may lead to the overfitting phenomenon. Therefore, we collected 3000 sets of data from 15 people who had never participated in the experiments and named this dataset Dataset 2. The new dataset is used to test the trained model. Table 5 shows the precision of the five SPR algorithms with new dataset. Table 6 shows the recall rate of the ISOM-SPR in comparison with the rest algorithms. Consistent with expected results, the precision and recall rate of all SPR algorithms have decreased. However, the results of ISOM-SPR are still the best of five SPR algorithms.

For the two data sets, the accuracy comparison of five SPR algorithms shows in Table 7. The accuracy of ISOM-SPR is the highest among all algorithms, regardless of Dataset 1 or Dataset 2. Consistent with expectations, test results are worse using Dataset 2 than Dataset 1, but the result of ISOM-SPR is still the best.

We conduct further analysis of ISOM-SPR; the confusion matrix of ISOM-SPR using Dataset 1 is shown in Table 8, while Dataset 2 is shown in Table 9. The precision and recall rates of all results using Dataset 1 are above 90%. It is clear from Table 8 and Table 9 that the identification results of LC and RC are the best, the result of LL’s misidentification is mostly LC, while the result of LR’s misidentification is mostly RC. The identification of WB and SS are the worst, with the misidentification of WB is mostly SS, while WB is always mistaken for SS.

## 5. Conclusions

In this paper, we designed a real-time sitting posture recognition system using flexible array pressure sensor and improved SOM classification method. The analysis and monitoring software can display the sitting posture pressure values and distributions, identify whether the sitting position is appropriate, and upload the data to the Cloud platform. The results of six kinds of sitting posture classification verified that the accuracy of ISOM-SPR reaches 95.67%, much better than DT-SPR, KM-SPR, BP-SPR, and SOM-SPR methods. The whole system has a great performance in using ISOM-SPR algorithm. High accuracy recognition of sitting posture can help users better check their sitting habits, so that users can consciously change some of their bad behaviors.

In future work, the number of samples can be further increased, in this way, the network model will have better applicability.

## Figures and Tables

**Table 1:** SOM-SPR and ISOM-SPR parameters.

Parameter	Symbol	Optimal Value
Number of the output neurons	*N*	6 (2 × 3)
Initial learning rate	*η*_0_	0.6
The time constant of the learning rate	*τ_η_*	1000
Learning gain	*σ*_0_	10
The time constant of the exponential function	*τ_σ_*	2
Initial neighborhood size	*N_c_*(0)	3
Total sample number	*M*	3600
The degree of differentiation	*r_min_*	0.15

**Table 2:** Multi-class confusion matrix for “left cross-legs”.

Actual	Predicted					
	LC	RC	LL	LR	WB	SS
LC	*TP*	*FN*	*FN*	*FN*	*FN*	*FN*
RC	*FP*	*TN*	*TN*	*TN*	*TN*	*TN*
LL	*FP*	*TN*	*TN*	*TN*	*TN*	*TN*
LR	*FP*	*TN*	*TN*	*TN*	*TN*	*TN*
WB	*FP*	*TN*	*TN*	*TN*	*TN*	*TN*
SS	*FP*	*TN*	*TN*	*TN*	*TN*	*TN*

**Table 3:** The precision of five SPR algorithms for Dataset 1.

Sitting Posture	DT-SPR	KM-SPR	BP-SPR	SOM-SPR	ISOM-SPR
LC	92.47%	96.77%	96.26%	94.94%	**97.40%**
RC	91.69%	96.62%	95.59%	93.17%	**97.88%**
LL	83.47%	93.15%	90.59%	93.50%	**96.47%**
LR	89.55%	93.27%	90.51%	92.65%	**95.89%**
WB	72.99%	87.89%	87.72%	89.66%	**91.60%**
SS	77.40%	91.41%	88.76%	90.60%	**94.58%**
Ave.	84.60%	93.19%	91.57%	92.42%	**95.64%**
Std.	0.0325	0.0056	0.0063	**0.0019**	0.0026

**Table 4:** The recall rate of five SPR algorithms for Dataset 1.

Sitting Posture	DT-SPR	KM-SPR	BP-SPR	SOM-SPR	ISOM-SPR
LC	94.17%	**100.00%**	94.33%	**100.00%**	**100.00%**
RC	95.67%	**100.00%**	93.83%	**100.00%**	**100.00%**
LL	84.17%	95.17%	94.67%	93.50%	**95.67%**
LR	85.67%	94.67%	93.83%	92.50%	**97.33%**
WB	75.67%	84.67%	84.50%	83.83%	**90.83%**
SS	72.50%	85.17%	88.17%	85.17%	**90.17%**
Ave.	84.64%	93.28%	91.56%	92.50%	**95.67%**
Std.	0.0442	0.0236	**0.0089**	0.0242	0.0094

**Table 5:** The precision of the five SPR algorithms for Dataset 2.

Sitting Posture	DT-SPR	KM-SPR	BP-SPR	SOM-SPR	ISOM-SPR
LC	90.31%	95.84%	91.50%	94.94%	**95.87%**
RC	89.31%	94.87%	91.53%	93.17%	**96.01%**
LL	82.39%	89.78%	87.74%	**93.50%**	93.00%
LR	85.67%	90.11%	87.98%	92.65%	**93.82%**
WB	72.03%	86.87%	88.81%	89.66%	**90.34%**
SS	79.15%	89.88%	88.38%	90.60%	**93.37%**
Ave.	83.14%	91.22%	89.32%	92.42%	**93.74%**
Std.	0.0236	0.0059	**0.0015**	0.0019	0.0022

**Table 6:** The recall rate of the five SPR algorithms for Dataset 2.

Sitting Posture	DT-SPR	KM-SPR	BP-SPR	SOM-SPR	ISOM-SPR
LC	93.17%	96.00%	93.33%	95.33%	**96.83%**
RC	94.67%	95.50%	93.67%	95.17%	**96.33%**
LL	82.67%	93.67%	91.83%	92.17%	**95.17%**
LR	83.67%	94.17%	91.50%	91.83%	**96.17%**
WB	73.83%	83.83%	82.00%	85.50%	**88.83%**
SS	71.50%	84.33%	83.67%	84.83%	**89.17%**
Ave.	83.25%	91.25%	89.33%	90.81%	**93.75%**
Std.	0.0456	0.0158	0.0132	0.0106	**0.0069**

**Table 7:** The accuracy of five SPR algorithms.

	DT-SPR	KM-SPR	BP-SPR	SOM-SPR	ISOM-SPR
Dataset 1	84.64%	93.28%	91.56%	92.50%	**95.67%**
Dataset 2	83.25%	91.25%	89.33%	90.81%	**93.75%**

**Table 8:** The confusion matrix of ISOM-SPR using Dataset 1.

Dataset 1	LC	RC	LL	LR	WB	SS	Total	Recall
LC	600	0	0	0	0	0	600	100.00%
RC	0	600	0	0	0	0	600	100.00%
LL	15	0	574	0	7	4	600	95.67%
LR	0	11	0	584	4	1	600	97.33%
WB	1	2	12	14	545	26	600	90.83%
SS	0	0	9	11	39	541	600	90.17%
Total	616	613	595	609	595	572		
Precision	97.40%	97.88%	96.47%	95.89%	91.60%	94.58%		

**Table 9:** The confusion matrix of ISOM-SPR using Dataset 2.

Dataset 1	LC	RC	LL	LR	WB	SS	Total	Recall
LC	581	0	16	0	3	0	600	96.83%
RC	0	578	0	18	4	0	600	96.33%
LL	18	0	571	0	10	1	600	95.17%
LR	0	18	0	577	5	0	600	96.17%
WB	3	6	11	10	533	37	600	88.83%
SS	4	0	16	10	35	535	600	89.17%
Total	606	602	614	615	590	573		
Precision	95.87%	96.01%	93.00%	93.82%	90.34%	93.37%		
